# Retraction: Effects of the Alexander technique on pain and adverse events in chronic non-specific neck pain: A systematic review and meta-analysis

**DOI:** 10.1371/journal.pone.0345732

**Published:** 2026-03-25

**Authors:** The PLOS One Editors

After this article [1] was published, concerns were raised about the methodological rigor and the clarity of reporting of this study. Specifically:

- In the Methods section several exclusion criteria were described but not justified, leaving the rationale for the study selection unclear.
- The meta-analysis reported in this article was conducted with three studies, including a quasi-randomized clinical trial with eight patients per group, despite substantial heterogeneity between the studies. This evidence base is too small and heterogeneous to support a meaningful or reliable meta-analysis.
- There appears to be discrepancy between the study blinding assessment reported for [2] in Table 2 in [1] and what has been reported by the authors of [2].
- The article contains repeated issues of unclear and inconsistent wording across the Abstract, Results, and Conclusion, which limits the reader’s ability to interpret the study’s findings accurately.

In addition, during the editorial reassessment of this article it was determined that the peer review did not meet the expected standards for *PLOS One*.

The corresponding author’s response did not resolve the journal’s concerns with the study reported in [1].

The *PLOS One* Editors retract this article [1] because it did not meet the journal’s third and fourth publication criteria [3]. We regret that the issues with the article were not addressed prior to its publication.

YW and YP did not agree with the retraction. DQ, YQ and LX either did not respond directly or could not be reached.
